# In vivo studies on antibiotic combination for the treatment of carbapenem-resistant Gram-negative bacteria: a systematic review and meta-analysis protocol

**DOI:** 10.1136/bmjos-2019-100055

**Published:** 2020-07-21

**Authors:** Elda Righi, Luigia Scudeller, Margherita Chiamenti, Kamilia Abdelraouf, Thomas Lodise, Elena Carrara, Alessia Savoldi, Dario Menghin, Gloria Pellizzari, Sally Ellis, Francois Franceschi, Laura Piddock, Chiara Rebuffi, Maurizio Sanguinetti, Evelina Tacconelli

**Affiliations:** 1 Infectious Diseases, Department of Diagnostics and Public Health, University of Verona, Verona, Italy; 2 Clinical Epidemiology and Biostatistics, IRCCS Ca’ Granda Ospedale Maggiore Policlinico di Milano Foundation, Milan, Italy; 3 Center for Anti-Infective Research and Development, Hartford Hospital, Hartford, Connecticut, USA; 4 Albany College of Pharmacy and Health Sciences, Albany, New York, USA; 5 Global Antibiotic Research & Development Partnership (GARDP), Geneva, Switzerland; 6 Fondazione IRCCS Policlinico San Matteo, Pavia, Italy; 7 Microbiology, A. Gemelli Hospital, Catholic University of the Sacred Heart, Rome, Italy

## Abstract

**Objective:**

There is poor evidence to determine the superiority of combination regimens versus monotherapy against infections due to carbapenem-resistant (CR) Gram-negative bacteria. In vivo models can simulate the pathophysiology of infections in humans and assess antibiotic efficacy. We aim to investigate in vivo effects of antibiotic combination on mortality and disease burden for infections due to CR *Acinetobacter baumannii*, *Pseudomonas aeruginosa* and Enterobacteriaceae and provide an unbiased overview of existing knowledge. The results of the study can help prioritising future research on the most promising therapies against CR bacteria.

**Methods and analysis:**

This protocol was formulated using the Systematic Review Protocol for Animal Intervention Studies (SYRCLE) Checklist. Publications will be collected from PubMed, Scopus, Embase and Web of Science. Quality checklists adapted by Collaborative Approach to Meta-Analysis and Review of Animal Data from Experimental Studies and SYRCLE’s risk of bias tool will be used. If the meta-analysis seems feasible, the ES and the 95% CI will be analysed. The heterogeneity between studies will be assessed by I^2^ test. Subgroup meta-analysis will be performed when possible to assess the impact of the studies on efficacy of the treatments. Funnel plotting will be used to evaluate the risk of publication bias.

**Dissemination:**

This systematic review and meta-analysis is part of a wider research collaboration project, the COmbination tHErapy to treat sepsis due to carbapenem-Resistant bacteria in adult and paediatric population: EvideNCE and common practice (COHERENCE) study that includes also the analyses of in vitro and human studies. Data will be presented at international conferences and the results will be published in peer-reviewed journals.

**PROSPERO registration number:**

CRD42019128104(available at: https://www.crd.york.ac.uk/prospero/display_record.php?ID=CRD42019128104).

**Strengths and limitations of this study**

- This is the first study aiming to summarise the in vivo evidence of antibiotic combination versus monotherapy to treat highly resistant (eg, carbapenem-resistant) infections in Gram-negative bacteria.
- The results of the study can help directing and prioritising future research on the most promising therapy against carbapenem-resistant bacteria.
- The heterogeneity of the studies may be high; however, we expect to include over 50 studies with at least 10–15 studies eligible for each Gram-negative bacterial species.
- Meta-analysis (and, if possible, network meta-analysis) and subgroup analysis will be performed to compare combination therapies and monotherapies.
- Quality of the studies will be systematically assessed according with Collaborative Approach to Meta-Analysis and Review of Animal Data from Experimental Studies and Systematic Review Protocol for Animal Intervention Studies’s risk of bias tools.

## Introduction

### Dimension of the problem: limited options for carbapenem-resistant infections

Infections caused by multidrug-resistant (MDR) Gram-negative bacteria (GNB) are associated with increased mortality compared with those caused by their susceptible counterpart.^1^ The increase of MDR infections, coupled with a limited number of novel antibiotics, has recently generated a significant unmet global medical need.^2^ An increase in resistance to carbapenems, considered as agents of *last resort* for MDR GNB severe infections, has become an urgent global health threat to address.^3^ In 2017, WHO published a priority pathogens list aimed at guiding research and development of new antibiotics.^4^ Critical pathogens included carbapenem-resistant (CR) *Acinetobacter baumannii*, *Pseudomonas aeruginosa* and Enterobacteriaceae. Although new drugs showing in vitro activity against CR GNB have recently become available, their use in real-world studies remains limited, and the optimal treatment for CR infections has yet to be established.^5^ Combination therapy, defined as the association of two or more antibiotics, is frequently used in clinical practice to treat CR *A. baumannii*, *P. aeruginosa* and *Klebsiella pneumoniae* infections. Aims of combination therapy include: (1) prevention of selection of drug-resistant strains; (2) maximisation of in vivo bacterial killing using antibiotics that act synergistically; (3) expansion of the antimicrobial spectrum of antibiotics. To date, however, the superiority of combination therapy compared with monotherapy has not been clearly demonstrated, and the impact of drug synergy on the evolution of resistance remains unclear. Moreover, increased toxicity can derive from the combination of antibiotics, and their efficacy may vary according to the pathogen species.

### Animal models to study combination therapy in CR infections

Provided that appropriate models are used according to the research objective^6^ and the associated limitations are clearly recognised,^7^ in vivo animal models represent valuable tools for testing activity, pharmacokinetics (PK) and toxicity of single antibiotics and their combinations.^8^ The capability of in vivo studies to investigate the host immune response, inoculum of bacteria at the infected site and antibiotic pharmacodynamics (PD) adds a dimension to in vitro testing and is essential to translate preclinical data into clinical trials. Animal models have served as a platform for preclinical assessments of novel antibiotics against CR bacteria.^9^ Furthermore, they help to investigate strategies to enhance antibiotic activities and overcome bacterial resistance.^8^ Various animal models (eg, murine, other rodents or vertebrates) are used to simulate the pathophysiology of MDR GNB infections and/or the antibiotic exposures observed in humans (table 1).

**Table 1:** Main types of animal models for the study of treatment efficacy in carbapenem-resistant bacteria and associated advantages or limitations

Site of infection	Advantages	Disadvantages
Thigh	PK/PD parameters often studied; low cost; highly reproducible	Lack of relevance for other infections (eg, pneumonia); time consuming (tissue homogenisation and filtration required for viable counting)
Septicaemia	Simple endpoints (mortality), less time consuming; assessment of novel antibiotics	Number of bacteria in blood may not correspond to clinical outcome
Endocarditis	Study of conditions favouring antibiotic resistance; test response to bloodstream infection similar to septicaemia model; PK/PD parameters can be studied	Number of bacteria in blood may not correspond to clinical outcome
Urinary tract infection (UTI)	Useful to simulate human UTI; measures can be taken from kidney, blood, urine, other organs	Technical skills needed to establish infection; time consuming (tissue homogenisation and filtration required for viable counting)
Pneumonia	Relevant for respiratory infections	Technical skills needed for inoculation; time consuming (tissue homogenisation and filtration required for viable counting)

PK/PD, pharmacokinetics/pharmacodynamics.

Septicaemia models are relevant to study MDR GNB since bloodstream infections have been used to compare antibiotic combination with monotherapy in humans.^10 11^ These models use easy-to-assess endpoints (eg, survival rate and bacterial load, measured as colony forming unit (CFU) in the blood) and are used in preclinical assessment of novel antimicrobials.^12 13^ Other models can test combinations against CR pathogens due to their capability to assess antibiotic efficacy against bacterial strains with minimum inhibitory concentrations (MICs) above the recommended breakpoint concentrations that designate drug susceptibility or to simulate human antibiotic exposure and immune status.^14–17^ Animal bacterial endocarditis is often used for antibacterial PK/PD studies.^18^ Due to the connection between bacterial persistence within the cardiac vegetation and development of resistance,^19^ it is also relevant for the study of infection relapse following treatment. The murine thigh infection model can also assess antibiotic PK/PD indices (eg, T>MIC, area under the curve (AUC)/MIC or Cmax/MIC).^20–22^ Urinary tract infection models have also been used to investigate novel antibiotics against MDR GNB.^13^ Finally, animal models of lung infection can simulate human pneumonia, which has the highest mortality in CR GNB infections.^23^ To study the efficacy of antibiotic combinations, synergy between different drug combinations is usually determined by statistically significant survival rate or bacterial load reduction in the combination therapy compared with the most active single-drug regimen. To differentiate synergistic activity from an additive effect, some authors^24^ suggested analysing in vivo synergy as a significant bactericidal effect of the drug combination in comparison with the sum of the bactericidal effect of each agent alone.

Due to ethical, technical and economic issues, invertebrate models of infections including Zebrafish, *Galleria mellonella* and other invertebrates currently represent an attractive option to study MDR infections.^25^ *G. mellonella* (greater wax moth) larvae, in particular, are used to investigate antimicrobial efficacy against CR GNB due to favourable turnaround times, easy procedures and defined endpoints.^26 27^ Invertebrate models still have important limitations since they may not be representative of human pathophysiology and PK/PD assessments are not feasible; thus, their results require validation in vertebrates.

### Immunocompetent and neutropenic models

Animal models of infections often use immune suppression to reduce the potential impact of immune responses on the effect of antimicrobials. Immunosuppression may be also necessary to establish infections that cause disease in humans but not in animals that may be inherently resistant. Pathogens with limited virulence may establish viable infections only in neutropenic animals and require lower bacterial inoculum.^28 29^ In general, a reduction of the amount of drug necessary to achieve similar microbiological outcome (ie, 1-log kill) is necessary in non-neutropenic compared with neutropenic antibacterial models according to the type of antibiotic and bacteria. Immunocompetent models remain key to reproduce what can happen in clinical practice and can be used to study the impact of leucocytes on antimicrobial efficacy. Both in vivo immunocompetent and neutropenic models of infection have been used to study MDR bacteria; furthermore, the efficacy of monotherapy or antimicrobial combinations can be compared in the same study between immunocompetent and neutropenic animals.^30 31^

### PK/PD studies

The development of large-scale, randomised controlled trials enrolling patients with MDR GNB infections is often not feasible due to the limited number of patients with MDR infections. In 2017, the Food and Drug Administration (FDA) approved the Limited Population Antibacterial Drug pathway to facilitate regulatory approval from smaller clinical studies targeting urgent unmet medical needs.^32^ This pathway, similar to regulatory submission guidelines, acknowledges the relevance of solid preclinical PK/PD data to support the efficacy and safety of antibiotics in patients.^33 34^ Specifically, in vitro PK/PD studies coupled with animal infection models are key tools for directing the antibiotic development process. Animal models of MDR GNB infections integrate the need for optimisation of antibiotic use (eg, optimum dosing strategy) with information that cannot be obtained in vitro, allowing for: (1) preclinical assessment for antibiotics; (2) selection of dose and dosing intervals; (3) support for establishing in vitro susceptibility breakpoint concentrations; (4) evaluation of resistance to antibiotics. Design of both animal and clinical studies rely on PK/PD indices of antibacterial activity and the related values that are set to achieve different magnitudes of bacterial killing.^35^ Preclinical PK/PD models are key in clarifying exposure–response relationship and design dosage regimens that can be applied in humans, according to the site of the infection.^36^ Limitations in the use of in vivo PK/PD models include the differences in the PK properties of antibiotics compared with those in humans. Furthermore, there is a limited feasibility of performing prolonged administrations, especially in small animals.^35^ For these reasons, multiple doses are usually administered, and transient renal impairment in animals through administration of uranyl nitrite is used to prolong the antibiotic half-life. Finally, animal ethical concerns are associated with high bacterial inoculum (eg, ≥10^8^ CFU/mL) that may cause increased early mortality, although high bacterial loads could be useful to investigate development of in vivo drug-resistant mutants.^37^

## Aim

The aim of this analysis is to evaluate the impact of antibiotic combinations on infections due to CR (including carbapenemase-producing) Gram-negative bacilli, namely *A. baumannii*, *P. aeruginosa* and Enterobacteriaceae (especially *K. pneumoniae*) analysing the available evidence from preclinical studies.

### Systematic review questions

1. What is the effect of antibiotic combination therapy versus monotherapy on mortality in animal models for infections due to CR *A. baumannii*, *P. aeruginosa* and Enterobacteriaceae?
2. What is the effect of antibiotic combination therapy versus monotherapy on disease burden reduction in animal models for infections due to CR *A. baumannii*, *P. aeruginosa* and Enterobacteriaceae?
3. What is the effect of antibiotic combination therapy versus monotherapy on drug-resistance development in animal models for infections due to CR *A. baumannii*, *P. aeruginosa* and Enterobacteriaceae?

## Methods and analysis

The structure of this protocol was formulated using the Systematic Review Protocol for Animal Intervention Studies (SYRCLE) Checklist.^38^

### Literature databases

PubMed, Scopus, Embase and Web of Science databases will be selected. All search strings have been discussed with a qualified librarian. The choice of keywords is based on the combinations of terms for carbapenem resistance, Gram-negative bacilli/bacteria (specifically, Enterobacteriaceae, *K. pneumoniae*, *P. aeruginosa*, *A. baumannii*), and related terms (eg, carbapenem, animal, in vivo) and included, specifically:

(“Gram-Negative Bacteria”[Majr] OR “gram negative” OR klebsiella OR acinetobacter OR pseudomonas OR enterobacteriaceae) AND (carbapenem* OR “carbapenem resistant” OR “carbapenem resistance” OR “multi drug resistant” OR “multi drug resistance” OR “pan drug resistant” OR “pan drug resistance” OR “extensive drug resistance” OR “extensive drug resistant”) AND (“in vivo” OR animal* OR murine).

Additional articles will be identified by manually searching the reference lists of included studies and relevant reviews related to the study question. Potentially eligible publications will be also searched on relevant conference proceedings of professional societies (Infectious Diseases Society of America (IDSA); European Society of Clinical Microbiology and Infectious Diseases) published between January 2016 and December 2018. Additionally, we will contact experts in the field.

### Study eligibility

Studies corresponding to PECOs (review questions for experimental animal exposure review) format will be selected.^39^ Population: all preclinical (animal) studies including infection models; Exposure: combination therapy for treatment of infections due to CR Enterobacteriaceae, *A. baumannii*, *P. aeruginosa*; Comparator: at least one intervention group in the study has to receive the combination of two (or more) antibiotics; Outcomes: (1) primary outcomes: proportion of animal mortality and/or reduction of disease burden (eg, change in the biomass based on culture results); (2) secondary outcomes: resistance development.

### Study selection procedure

The study selection procedure will include two screening phases with two observers per phase:

- Phase 1: articles retrieved from the databases will be screened based on title and abstract outlined in the search strategy by two independent researchers. In the case of at least one researcher opting for potential eligibility of the study, the full text will be retrieved.
- Phase 2: full text of the articles obtained after preliminary selection will be retrieved. Two researchers will independently assess the eligibility of full text for inclusion. Discrepancies during article screening will be resolved by consensus between the two reviewers. Disagreement between them over the eligibility of particular studies will be resolved through discussion with a third reviewer.

### Study selection criteria

Inclusion criteria related to the type of study design, study population and disease model, the type of intervention and the outcome measures will be applied to the systematic review.

#### Type of study design, animals and disease model, intervention

All types of studies describing the effect of combination therapy against CR infections due to *A. baumannii*, *P. aeruginosa* and Enterobacteriaceae will be included. Reviews, editorials and protocol papers will be excluded.

All animal species, regardless of age and sex, will be included. All models of CR infections due to *A. baumannii*, *P. aeruginosa* and Enterobacteriaceae will be considered. Studies testing disseminated infections and/or measuring bacterial growth from blood will be included.

We will consider as type of intervention any antibiotic combination therapy, defined as the association of two or more antibiotics, including that of an antibiotic with a beta-lactam beta-lactamase (BLBLI) combination treatment and dual beta-lactam therapy (eg, dual carbapenem therapy). Studies with any route, dose and treatment schedule for antibiotic administration will be eligible for inclusion. Single antibiotic (monotherapy, including BLBLIs) will be considered as comparator. When applicable, comparison between combination therapies will be performed; network meta-analysis (NMA) will be considered if more than one study will have at least one treatment arm in common to infer.

A list of antibiotics that can be used in combination against CR bacteria in clinical practice is reported in table 2. In the majority of cases, we expect to analyse combination therapies versus monotherapy with a carbapenem, colistin, an aminoglycoside (amikacin, gentamicin or tobramycin) or tigecycline.

**Table 2:** List of principal antibiotics used as monotherapy or in combination against carbapenem-resistant bacilli in clinical practice (already approved or in late stage of development)

Class	Approved	Recently* FDA approved	Phase 3 studies
Aminoglycoside	Gentamicin; amikacin tobramycin	Plazomicin	
BLBLI	Ampicillin–sulbactam Piperacillin–tazobactam Cefoperazone–sulbactam	Ceftadizime–avibactam Meropenem–vaborbactam Ceftolozane–tazobactam Imipenem–relebactam	Aztreonam–avibactam Ceftaroline–avibactam Cefepime–taniborbactam Cefepime–zidebactam
Carbapenem	Meropenem; imipenem; doripenem; ertapenem		
Cephalosporin			Cefiderocol
Dihydrofolate reductase inhibitor			Iclaprim
Fluoroquinolone		Delafloxacin	
Monobactam	Aztreonam		
Phosphonic acid derivative	Fosfomycin		
Polymyxin	Polymyxin B polymyxin E/colistin		
Rifamycin	Rifampicin		
Tetracycline	Doxycycline; tigecycline; minocycline	Eravacycline Omadacycline	

BLBLI, beta-lactam/beta-lactamase inhibitors; FDA, Food and Drug Administration.

Exclusion criteria for type of intervention will be:

- Studies that do not include at least one intervention group receiving combination therapy.
- Treatment with antibiotics that are not approved for treatment by FDA and/or European Medicines Agency or antibiotics that are not in late stage of clinical development for use in clinical practice (eg, phase 3 studies).

#### Outcome measures

Studies will be included if they report separately for each treatment regimen (combination or monotherapy or blank) group at least one among: (1) animal mortality rate and proportion measured at completion of study; (2) extent of bacterial burden reduction based on culture results (total bacterial counts measured as change in the biomass, eg, CFU/g or mL) timed at completion of study. Studies will be excluded if the relevant outcome measures cannot be obtained through extraction. Whenever possible, data extraction from graphs will be attempted if no raw data are provided.

#### Protocol restrictions

We did not include any language restrictions in our search. If an English language version is not available, abstracts and full text that are relevant to the review will be translated into English. Studies published from 1 January 1985 until 31 December 2018 will be included. In case data or experimental groups were used repeatedly in more than one study, for example, to address various hypotheses, data will be included only once.

**Figure 1:**
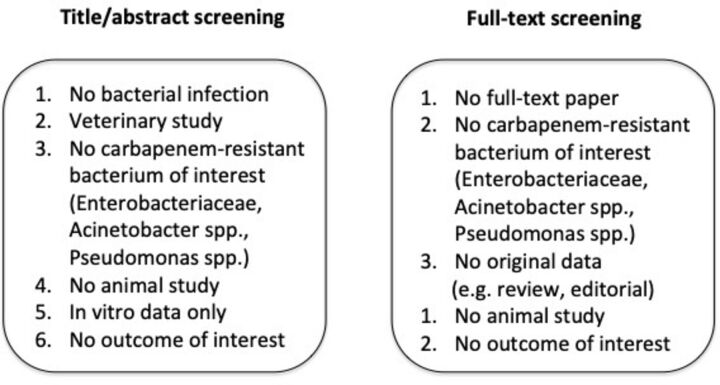
Exclusion criteria order of priority per screening phase.

Criteria order of priority according to the screening phase is reported in figure 1.

### Study characteristics to be extracted and data extraction

Data will be extracted from either text or tables in the Results section of the manuscripts of interest. We will pilot test and use a standardised Microsoft Excel form to extract data regarding PECOs questions, including study characteristics, animal model of infection, antibiotic treatment and outcomes of interest. Once the database for data extraction is piloted and validated, two reviewers will independently complete data extraction. In case the control group serves as comparison for multiple treatment groups, the number of animals reported in the control group will be divided by the number of treatment groups served in order to control for multiple comparisons when weighting of effect size. Categorical variables within study results will be entered as proportions (eg, proportion of animals surviving) using a defined coding system in an electronic database. Continuous variables will be expressed as mean (normally distributed data), or median (non-normally distributed data; eg, median survival in days). Back calculation of the necessary data will be allowed.

#### Parameters for data extraction

##### Study ID

For full-text papers, digital object identifier, first author, journal, publication year, source of funding will be retrieved. For conference proceedings, first author, conference, publication year and source of funding will be extracted.

##### Animal models

The following data will be extracted: animal type (eg, non-vertebrates, eg, *Galleria*; rodent or non-rodent), species, breed or strain; sex, age, weight; immunocompetent or immunocompromised status, uranyl nitrate administration.

##### Infection model

Data to be extracted will include: site of inoculum (intraperitoneal, tail injection, inhalation, thigh injection, urethral injection, other), positivity of blood for bacteria, type of infection (eg, sepsis and/or peritonitis, lung, thigh, urinary tract infection, other), type of bacteria (origin, eg, biobank vs clinical sample, strain type, molecular mechanism of resistance, MICs); mortality among untreated animals (controls).

##### Intervention characteristics

Type of antibiotic administered, time of delivery relative to time of infection, loading and maintenance dose, administration schedule, PK/PD indices (eg, T>MIC, AUC/MIC or C_max_/MIC), simulated human antibiotic exposure, duration of treatment and duration of follow-up will be extracted. In case of multiple time points, the latest one available (eg, end of study) will be included for consistency and because of its relevance for clinical implications.

#### Outcome measurements

##### Quantitative data

Mortality rate will be expressed as percentage of dead animals at end of study. Reduction of infection burden will be quantified according to the type of infection model by subtracting bacterial load (expressed as CFU per tissue or mL) at end of treatment (EOT) from that of the untreated (control) mice at time 0 or EOT.

We expect to retrieve outcomes mainly from the first time point following completion of therapy administration, while a limited number of studies will include long-term bacterial infections (eg, models with untreated infections that can last for weeks) allowing to analyse outcomes at multiple timepoints (eg, short-term and long-term mortality).

##### Qualitative data

Absence of bacterial growth at end of study (yes/no), reduction of infection burden with combination therapy versus monotherapy and emergence of resistant mutants at 24–48 hours (bacterial growth in the presence of drug, yes/no) will be also collected.

##### Data analysis

To assess the efficacy of combination treatment, mortality rate and/or bacterial load reduction in the combination therapy arm will be compared with the most active single-drug regimen. According to the number of studies available, different combination regimens will be compared. We will analyse and compare combination regimens that can be used in clinical practice according to the evidence provided by available reports or recommendations.

## Risk-of-bias assessment

No clear consensus to assess the methodology and potential biases of animal studies, including those reporting toxicology results—that are essential to evaluate the damage from exposure to environmental chemicals or drug safety prior to human testing—is available.^40^ Various tools are used and, of these, only a minority has been tested for reliability or validity.^41 42^ We will use SYRCLE’s tool for assessing risk of bias based on 10 domains including selection, performance, detection, attrition and reporting biases as summarised in box 1.^43^ A specific part of the Collaborative Approach to Meta-Analysis and Review of Animal Data from Experimental Studies Checklist will be used to detect additional risk of bias^42 44^ including four additional questions (box 1). Finally, to assess the absence of bias related to the use of antibiotics and bacterial strains in the infection model, an adapted version of the criteria for reporting and evaluating ecotoxicity data tool for animal studies will be used assessing seven elements,^45^ and four additional elements applying specifically to infection models will be included (box 1). Publications will receive a point for compliance of each item in the checklist from which group median scores will be calculated. Data not reported will score 0 in the checklist.

Box 1 **Questions included in the risk-of-bias assessment**

1. Was the allocation sequence adequately generated and applied?

2. Were the groups similar at baseline or were they adjusted for confounders in the analysis?

3. Was the allocation adequately concealed?

4. Were the animals randomly housed during the experiment?

5. Were the caregivers and/or investigators blinded from knowledge which intervention each animal received during the experiment?

6. Were animals selected at random for outcome assessment?

7. Was the outcome assessor blinded?

8. Were incomplete outcome data adequately addressed?

9. Are reports of the study free of selective outcome reporting?

10. Was the study apparently free of other problems that could result in high risk of bias?

11. Was the publication reported in a peer-reviewed journal?

12. Was a sample size calculation and/or a power calculation method reported?

13. Was compliance with animal welfare regulations reported?

14. Were potential conflicts of interest reported?

15. Are the antibiotics used identified clearly with name?

16. Is the source of the antibiotic trustworthy?

17. Are the microorganisms well described (eg, name, growth, strain)?

18. Is the experimental system described for the microorganism (eg, choice of medium for bacterial growth)?

19. Are frequency and duration of exposure as well as timepoints of observations explained?

20. Are the study endpoints and their methods of determination clearly described?

21. Is the number of replicates (or complete repetitions of experiment) given?

22. Was the presence of bacteria assessed in blood in sepsis models?

23. Was mortality reported in untreated controls?

24. Were drug concentrations in blood assessed in at least one arm?

25. Was mortality tested as outcome in sepsis models (eg, vs clearance of infection from blood)?

## Data analysis

### Strategy for data synthesis and meta-analysis

We plan to conduct a quantitative synthesis through meta-analysis if conditions apply. Data will be combined in a systematic review, forest plot and subsequent meta-analysis. For PECO where quantitative synthesis will not be feasible, available data obtained from the included studies will be summarised in terms of PECO questions, overall study conclusions and risk of bias. If the comparability of experimental conditions will allow it, we will perform an NMA. Statistical analyses will be performed using Stata V.15 or higher. We expect to include over 50 studies with at least 10 studies eligible for each GNB species.

#### Effect measure

We expect to face significant differences in effect between non-vertebrates (eg, *Galleria*) and vertebrates (eg, rodents) and non-rodents (eg, large animals) for our analyses. Therefore, we will stratify these groups upfront and pool these data separately for additional meta-regression analyses. We expect a limited number of studies reporting monotherapy at different doses, since the review will include articles comparing treatments rather than studies on the efficacy of a specific antibiotic for registration purposes. If multiple doses are provided, pooled results will be analysed and, if applicable, data analyses according to the dose used (eg, low, standard, high dose) will be performed. For the other parameters, we expect outcome measures to be consistent and generally used in the same way, although differences may apply to different infection models (eg, sepsis, thigh infection, pneumonia, etc). Results will be analysed through a pooled outcomes meta-analysis.

Pooled effect size will be expressed as relative risks for mortality compared with monotherapy and as standardised mean difference for reduction in bacterial burden. All data will be calculated with the corresponding 95% CI. When necessary, medians and IQR will be used to estimate means and SDs (and SEs). Monotherapy will be considered as comparator for combination therapies, while comparison with no therapy (eg, saline) will not be included.

#### Effect models

If conditions apply, results will be pooled via fixed (low or fair heterogeneity) or random (moderate) effect meta-analysis.

#### Heterogeneity

Statistical heterogeneity among studies will be measured by I^2^ test and will be considered as follows: 0–0.25 low, 0.25–0.5 fair, 0.5–0.75 moderate and >0.75 high. Any reduction in heterogeneity will be assessed through subgroup analysis and meta-regression analysis for categorical and continuous variables, respectively.

#### Analysis of subgroups or subsets

If a sufficient number of studies (eg, at least 2) are eligible for inclusion in the meta-analysis, a subgroup analysis will be performed to analyse the effect of combination versus monotherapy according to: (1) bacteria species (eg, *A. baumannii*, *P. aeruginosa, K. pneumoniae* or other Enterobacteriaceae); (2) animal model, including site of infection (eg, sepsis, thigh infection, pneumonia, etc) and immunocompromised or immunocompetent status; (3) type of inoculum (eg, low or high inoculum infections); (4) molecular mechanisms of carbapenem resistance. In these subgroup analyses, Bonferroni correction will be applied for p values and 95% CI.

#### Sensitivity and publication bias

Sensitivity analyses excluding one study each time and recalculating the combined results will be completed to investigate the influence of an individual dataset on the pooled data.

Funnel plots will be generated to explore the possibility of publication bias.

## Conclusions and clinical implications

Since in vivo models can simulate the pathophysiology of infections in humans and assess antibiotic efficacy, the results of the study can help prioritising future research on the most promising therapies against CR bacteria and inform clinical trials in humans. Furthermore, the study of clinical practice implications of combination therapy against CR GNB is part of the wider project funded by Global Antibiotic Research and Development Partnership/WHO, the COmbination tHErapy to treat sepsis due to carbapenem-Resistant bacteria in adult and paediatric population: EvideNCE and common practice (COHERENCE) study. The COHERENCE project includes also the analysis of in vitro and human studies.

## References

[1] Holmberg SD, Solomon SL, Blake PA. Health and economic impacts of antimicrobial resistance. Rev Infect Dis 1987;9:1065–78. 10.1093/clinids/9.6.1065 3321356

[2] Boucher HW, Talbot GH, Bradley JS, et al. Bad bugs, no drugs: no ESKAPE! an update from the infectious diseases Society of America. Clin Infect Dis 2009;48:1–12. 10.1086/595011 19035777

[3] Centers for Disease Control and Prevention (CDC). Antibiotic resistance threats in the United States, 2013. Available: https://www.cdc.gov/drugresistance/threat-report-2013/pdf/ar-threats-2013-508.pdf

[4] Tacconelli E, Carrara E, Savoldi A, et al. Discovery, research, and development of new antibiotics: the who priority list of antibiotic-resistant bacteria and tuberculosis. Lancet Infect Dis 2018;18:318–27. 10.1016/S1473-3099(17)30753-3 29276051

[5] Peri AM, Doi Y, Potoski BA, et al. Antimicrobial treatment challenges in the era of carbapenem resistance. Diagn Microbiol Infect Dis 2019;94:413–25. 10.1016/j.diagmicrobio.2019.01.020 30905487

[6] Swearengen JR. Choosing the right animal model for infectious disease research. Animal Model Exp Med 2018;1:100–8. 10.1002/ame2.12020 30891554 PMC6388060

[7] Zak O, O'Reilly T. Animal models in the evaluation of antimicrobial agents. Antimicrob Agents Chemother 1991;35:1527–31. 10.1128/AAC.35.8.1527 1929323 PMC245213

[8] Baker SJ, Payne DJ, Rappuoli R, et al. Technologies to address antimicrobial resistance. Proc Natl Acad Sci U S A 2018;115:12887–95. 10.1073/pnas.1717160115 30559181 PMC6304975

[9] Andes D, Craig WA. Animal model pharmacokinetics and pharmacodynamics: a critical review. Int J Antimicrob Agents 2002;19:261–8. 10.1016/S0924-8579(02)00022-5 11978497

[10] Qureshi ZA, Paterson DL, Potoski BA, et al. Treatment outcome of bacteremia due to KPC-producing Klebsiella pneumoniae: superiority of combination antimicrobial regimens. Antimicrob Agents Chemother 2012;56:2108–13. 10.1128/AAC.06268-11 22252816 PMC3318350

[11] Tumbarello M, Viale P, Viscoli C, et al. Predictors of mortality in bloodstream infections caused by Klebsiella pneumoniae carbapenemase-producing K. pneumoniae: importance of combination therapy. Clin Infect Dis 2012;55:943–50. 10.1093/cid/cis588 22752516

[12] Drusano GL. Antimicrobial pharmacodynamics: critical interactions of 'bug and drug'. Nat Rev Microbiol 2004;2:289–300. 10.1038/nrmicro862 15031728

[13] Reyes N, Aggen JB, Kostrub CF. In vivo efficacy of the novel aminoglycoside ACHN-490 in murine infection models. Antimicrob Agents Chemother 2011;55:1728–33. 10.1128/AAC.00862-10 21282439 PMC3067188

[14] Abdelraouf K, Kim A, Krause KM, et al. In Vivo Efficacy of Plazomicin Alone or in Combination with Meropenem or Tigecycline against Enterobacteriaceae Isolates Exhibiting Various Resistance Mechanisms in an Immunocompetent Murine Septicemia Model. Antimicrob Agents Chemother 2018;62:e01074–18. 10.1128/AAC.01074-18 29866866 PMC6105799

[15] Cai Y, Yang D, Wang J, et al. Activity of colistin alone or in combination with rifampicin or meropenem in a carbapenem-resistant bioluminescent Pseudomonas aeruginosa intraperitoneal murine infection model. J Antimicrob Chemother 2018;73:456–61. 10.1093/jac/dkx399 29149302

[16] Fan B, Guan J, Wang X, et al. Activity of colistin in combination with meropenem, tigecycline, fosfomycin, fusidic acid, rifampin or sulbactam against extensively drug-resistant Acinetobacter baumannii in a murine thigh-infection model. PLoS One 2016;11:e0157757. 10.1371/journal.pone.0157757 27315107 PMC4912081

[17] Pachón-Ibáñez ME, Fernández-Cuenca F, Docobo-Pérez F, et al. Prevention of rifampicin resistance in Acinetobacter baumannii in an experimental pneumonia murine model, using rifampicin associated with imipenem or sulbactam. J Antimicrob Chemother 2006;58:689–92. 10.1093/jac/dkl303 16870647

[18] Andes DR, Craig WA. Pharmacodynamics of fluoroquinolones in experimental models of endocarditis. Clin Infect Dis 1998;27:47–50. 10.1086/514624 9675448

[19] Durack DT, Beeson PB. Experimental bacterial endocarditis. II. survival of a bacteria in endocardial vegetations. Br J Exp Pathol 1972;53:50–3. 4111329 PMC2072380

[20] Bulik CC, Nicolau DP, Berkhout J, Melchers MJ, van Mil AC. In vivo efficacy of simulated human dosing regimens of prolonged-infusion doripenem against carbapenemase- producing Klebsiella pneumoniae. Antimicrob Agents Chemother 2010;54:4112–5. 10.1128/AAC.00026-10 20660688 PMC2944615

[21] Craig WA, Redington J, Ebert SC. Pharmacodynamics of amikacin in vitro and in mouse thigh and lung infections. J Antimicrob Chemother 1991;27 Suppl C:29–40. 10.1093/jac/27.suppl_C.29 1830302

[22] Vogelman B, Gudmundsson S, Leggett J, et al. Correlation of antimicrobial pharmacokinetic parameters with therapeutic efficacy in an animal model. J Infect Dis 1988;158:831–47. 10.1093/infdis/158.4.831 3139779

[23] King M, Heil E, Kuriakose S, et al. Multicenter study of outcomes with ceftazidime-avibactam in patients with carbapenem-resistant Enterobacteriaceae infections. Antimicrob Agents Chemother 2017;61:e00449–17. 10.1128/AAC.00449-17 28483952 PMC5487633

[24] Fantin B, Carbon C. In vivo antibiotic synergism: contribution of animal models. Antimicrob Agents Chemother 1992;36:907–12. 10.1128/AAC.36.5.907 1510412 PMC188745

[25] Neely MN. The zebrafish as a model for human bacterial infections. Methods Mol Biol 2017;1535:245–66. 10.1007/978-1-4939-6673-8_16 27914084

[26] Hornsey M, Wareham DW. In vivo efficacy of glycopeptide-colistin combination therapies in a Galleria mellonella model of Acinetobacter baumannii infection. Antimicrob Agents Chemother 2011;55:3534–7. 10.1128/AAC.00230-11 21502628 PMC3122470

[27] Peleg AY, Jara S, Monga D, et al. Galleria mellonella as a model system to study Acinetobacter baumannii pathogenesis and therapeutics. Antimicrob Agents Chemother 2009;53:2605–9. 10.1128/AAC.01533-08 19332683 PMC2687231

[28] Lorian V. Chapter 15: evaluation of antimicrobials in experimental animal infections. In: Antibiotics in laboratory medicine. 5 edn. Philadelphia: Lippincott Williams & Wilkins, 2005.

[29] McConnell MJ, Actis L, Pachón J. Acinetobacter baumannii: human infections, factors contributing to pathogenesis and animal models. FEMS Microbiol Rev 2013;37:130–55. 10.1111/j.1574-6976.2012.00344.x 22568581

[30] Hagihara M, Crandon JL, Urban CM, et al. KPC presence in Pseudomonas aeruginosa has minimal impact on the in vivo efficacy of carbapenem therapy. Antimicrob Agents Chemother 2013;57:1086–8. 10.1128/AAC.01748-12 23254422 PMC3553679

[31] Wiskirchen DE, Crandon JL, Nicolau DP. Impact of various conditions on the efficacy of dual carbapenem therapy against KPC-producing Klebsiella pneumoniae. Int J Antimicrob Agents 2013;41:582–5. 10.1016/j.ijantimicag.2013.02.015 23611306

[32] U.S. Department of Health and Human Services - Food and Drug Administration (FDA), Center for Drug Evaluation and Research (CDER), Center for Biologics Evaluation and Research (CBER). Limited population pathway for antibacterial and antifungal drugs guidance for industry (draft guidance, 2018.

[33] European Medicines Agency (EMA). Committee for medicinal products for human use (CHMP). 2016. guideline on the use of pharmacokinetics and pharmacodynamics in the development of antimicrobial medicinal products, 2015.

[34] Bhavnani SM, Zhang L, Hammel JP, et al. Pharmacokinetic/Pharmacodynamic target attainment analyses to support intravenous and oral lefamulin dose selection for the treatment of patients with community-acquired bacterial pneumonia. J Antimicrob Chemother 2019;74:iii35–41. 10.1093/jac/dkz089 30949705 PMC6449570

[35] Velkov T, Bergen PJ, Lora-Tamayo J, et al. PK/PD models in antibacterial development. Curr Opin Microbiol 2013;16:573–9. 10.1016/j.mib.2013.06.010 23871724 PMC3834155

[36] Louie A, Liu W, Fikes S, et al. Impact of meropenem in combination with tobramycin in a murine model of Pseudomonas aeruginosa pneumonia. Antimicrob Agents Chemother 2013;57:2788–92. 10.1128/AAC.02624-12 23571540 PMC3716130

[37] Drusano GL, Johnson DE, Rosen M, et al. Pharmacodynamics of a fluoroquinolone antimicrobial agent in a neutropenic rat model of Pseudomonas sepsis. Antimicrob Agents Chemother 1993;37:483–90. 10.1128/AAC.37.3.483 8384815 PMC187696

[38] de Vries RBM, Hooijmans CR, Langendam MW, et al. A protocol format for the preparation, registration and publication of systematic reviews of animal intervention studies. Evid Based Preclin Med 2015;2:e00007–9. 10.1002/ebm2.7

[39] NTP (National Toxicology Program). Handbook for conducting a Literature-Based health assessment using office of health assessment and translation (OHAT) approach for systematic review and evidence integration, 2015. Available: http://ntp.niehs.nih.gov/go/38673

[40] Krauth D, Woodruff TJ, Bero L. Instruments for assessing risk of bias and other methodological criteria of published animal studies: a systematic review. Environ Health Perspect 2013;121:985–92. 10.1289/ehp.1206389 23771496 PMC3764080

[41] Hobbs DA, Warne MSJ, Markich SJ. Evaluation of criteria used to assess the quality of aquatic toxicity data. Integr Environ Assess Manag 2005;1:174–80. 10.1897/2004-003R.1 16639883

[42] Sena E, van der Worp HB, Howells D, Macleod M, et al. How can we improve the pre-clinical development of drugs for stroke? Trends Neurosci 2007;30:433–9. 10.1016/j.tins.2007.06.009 17765332

[43] Hooijmans CR, Rovers MM, de Vries RBM, et al. SYRCLE's risk of bias tool for animal studies. BMC Med Res Methodol 2014;14:43. 10.1186/1471-2288-14-43 24667063 PMC4230647

[44] Macleod MR, Lawson McLean A, Kyriakopoulou A, et al. Risk of bias in reports of in vivo research: a focus for improvement. PLoS Biol 2015;13:e1002273. 10.1371/journal.pbio.1002273 26460723 PMC4603955

[45] Moermond CTA, Kase R, Korkaric M, et al. CRED: criteria for reporting and evaluating ecotoxicity data. Environ Toxicol Chem 2016;35:1297–309. 10.1002/etc.3259 26399705

